# Retrieval and Reconsolidation Accounts of Fear Extinction

**DOI:** 10.3389/fnbeh.2016.00089

**Published:** 2016-05-09

**Authors:** Ravikumar Ponnusamy, Irina Zhuravka, Andrew M. Poulos, Justin Shobe, Michael Merjanian, Jeannie Huang, David Wolvek, Pia-Kelsey O’Neill, Michael S. Fanselow

**Affiliations:** Departments of Psychology, Psychiatry and Biobehavioral Sciences, and The Brain Research Institute, University of CaliforniaLos Angeles, CA, USA

**Keywords:** fear, extinction, reconsolidation, consolidation, memory, anxiety disorders

## Abstract

Extinction is the primary mode for the treatment of anxiety disorders. However, extinction memories are prone to relapse. For example, fear is likely to return when a prolonged time period intervenes between extinction and a subsequent encounter with the fear-provoking stimulus (spontaneous recovery). Therefore there is considerable interest in the development of procedures that strengthen extinction and to prevent such recovery of fear. We contrasted two procedures in rats that have been reported to cause such deepened extinction. One where extinction begins before the initial consolidation of fear memory begins (immediate extinction) and another where extinction begins after a brief exposure to the consolidated fear stimulus. The latter is thought to open a period of memory vulnerability similar to that which occurs during initial consolidation (reconsolidation update). We also included a standard extinction treatment and a control procedure that reversed the brief exposure and extinction phases. Spontaneous recovery was only found with the standard extinction treatment. In a separate experiment we tested fear shortly after extinction (i.e., within 6 h). All extinction procedures, except reconsolidation update reduced fear at this short-term test. The findings suggest that strengthened extinction can result from alteration in both retrieval and consolidation processes.

## Introduction

Fear extinction creates a new “safe” memory that co-exists with the original fear memory (Bouton, 1993). Because the two memories are retrieved by the same cue, extinction presents a retrieval problem because it is not clear which memory will be retrieved in any given situation. Retrieval of the fear memory leads to an undesired return of fear. For example, fear spontaneously recovers when substantial time intervenes between extinction and testing. Return of fear contributes to relapse following exposure-based therapies (Bruce et al., 2005), establishing a need for methods capable of making extinction robust against fear recovery.

Myers et al. (2006) hypothesized that beginning extinction soon after fear acquisition might be such a procedure. Memories undergo time-dependent consolidation and they reasoned that if extinction occurred before consolidation of the fear memory, it would be erased and unrecoverable. Supporting this hypothesis, there was less fear recovery if extinction occurred 1 h, rather than 24 h after acquisition. While Myers et al. (2006) attributed their findings to consolidation failure, a retrieval explanation is also possible. Having acquisition and extinction close in time might result in both memories being encoded into the same episode. If the extinction memory was dominant, retrieval of that episode should not provoke fear. Maren and Chang (2006) reported that when fear levels are very high during extinction there was little evidence of a long-term extinction memory. According to a retrieval interpretation, if acquisition and extinction are part of the same episode, and fear is high during that episode, then a fear memory would be retrieved. In contrast to Myers et al. (2006) and support of Maren and Chang (2006) many studies found failure of immediate extinction on fear memory [rats (Morris et al., 2005; Archbold et al., 2010), mice (Stafford et al., 2013) and human (Alvarez et al., 2007; Norrholm et al., 2008; Schiller et al., 2008; Huff et al., 2009)].

A practical limitation in using immediate extinction is the need to begin extinction close to the time of trauma. Monfils et al. (2009) suggested a way to ease this limitation, by providing a brief reminder to open a window of vulnerability before a typical experimental extinction that is done 24 h or longer after fear conditioning. The logic is that memories are vulnerable to amnestic agents both shortly after fear learning (Schafe and LeDoux, 2000) and after a reminder (Nader et al., 2000). Monfils et al. (2009) found that, indeed, extinction memories were robust against fear recovery when a reminder shortly preceded extinction (for opposite results in both rodents and humans, see Chan et al., 2010; Costanzi et al., 2011; Kindt and Soeter, 2013; Stafford et al., 2013). This effect has become known as reconsolidation update (Monfils et al., 2009). There are several possible explanations as to why this procedure works. One based on the consolidation and reconsolidation literatures is that reopening the “vulnerability window” allows the original fear memory to be deconsolidated in much the way a protein synthesis inhibition allows deconsolidation of the original memory. A second, which does not depend on reconsolidation mechanisms at all, suggests that the reminder allows the extinction learning to be incorporated into the original memory and thereby results in a change in the encoded CS-US relationship. It is difficult to reconcile this account of the more durable extinction result with what happens in traditional multi-trial extinction procedures as these also have reminders but do not produce enduring extinction. These two accounts of the reminder-extinction effect do make a differential prediction. The former deconsolidation account suggests that at a short-term test the original fear memory should be intact just as it is in traditional consolidation and reconsolidation experiments. The fear memory should only disappear after a longer-term test interval. The latter interpretation in terms of a degraded CS-US relationship, suggests no difference at a short- or long-term interval as the CS-US relationship would be degraded at either time point. Therefore, we conducted both long and short-term tests to distinguish these accounts.

This reconsolidation update effect is also interpretable from retrieval-based models (Bjork, 1994; Schmidt and Bjork, 1992), which predict that variability in retrieval practice makes memories more retrievable. According to their model, variation increases the storage strength of information to be learned by making retrieval of past learning easier via the availability of cues that were present during prior learning. The retrieval model stresses the importance of variability of exposure but the order of the session types is less important. Variability in extinction training conditions leads to enhanced extinction retrieval in studies of human fear memory (e.g., Rowe and Craske, 1998a,b). The retrieval and extinction sessions can be viewed as two different extinction experiences. This increased retrieval variability may render extinction memories more retrievable and therefore, more resistant to fear recovery. To test this interpretation, we used a procedure that retained the same variability of experience as the procedure thought to generate reconsolidation update but did not give extinction during a window of vulnerability. We simply gave our extinction session prior to the reminder. This lead to a five group design assessing spontaneous recovery following traditional massed extinction, immediate extinction, reconsolidation update and our variability control procedure. Note that our fear extinction—retrieval methods are similar to methods used by Baker et al. (2013) and Millan et al. (2013) that showed enhanced fear extinction retrieval in adolescent rats and enhanced extinction retrieval of alcohol seeking in adult rats respectively.

To further distinguish memory reconsolidation and retrieval accounts, Experiment 2 tested fear shortly after extinction (3.25 h) and at the typical 24 h period after extinction. When memories are tested while consolidation or reconsolidation processes are ongoing, amnestic manipulations typically have no effect. Rather, their effect emerges later when the memory is dependent on that earlier consolidation process (Nader et al., 2000; Schafe and LeDoux, 2000). If these manipulations affect memory consolidation then fear memory should be intact during the early test, but absent during the later test.

## Materials and Methods

### Animals

In the present study, we used male adult rats (Long Evans; HsdBlu:LE) initially weighing 250–280 g (Harlan, Indianapolis, IN, USA). After arrival at UCLA, the rats were housed individually in standard stainless steel cages on 12 h light/dark cycle and were provided free access to food and tap water. After being housed, the rats were handled daily (60–90 s per rat) for 7 days to acclimate them to the experimenter. All procedures conformed to the USA National Research Council Guide to the Care and Use of Laboratory Animals and were approved by the UCLA Institutional Animal Care and Use Committee. The number of animals used was the minimum required to ensure reliability of the results, and every effort was made to minimize animal discomfort while achieving the goals of the experiment.

### Behavioral Parameters

All behavioral training was performed using two sets of four identical fear conditioning chambers equipped with a Med-associates VideoFreeze near infrared video tracking system. Chambers were enclosed within sound attenuated chambers in a well-lit room separated from the observers.

### Contexts

Two contexts that differ on spatial location, odor, interior design (opaque or clear), background noise, lighting and transport were used. All groups were fear conditioned in context A. All retrieval/extinction and testing sessions occurred in context B. However, importantly, all statistical comparisons were made between groups that were tested in the same context after equivalent exposure to that context.

### Context A

The context A environment consisted of aluminum (side walls) and Plexiglas (front, back, and top) chambers (30 × 25 × 25 cm, Med-Associates, Inc. St. Albans, VT, USA) and two white plastic side walls (24 cm × 21 cm) placed at 60° to the floor, forming a triangular enclosure. The floor of each chamber had 18 stainless steel rods (4 mm diameter, 1.5 cm apart) connected to a shock scrambler and generator (which, along with internal ventilation fans, supplied background noise of 60 dB, A scale). The context A chambers were cleaned with 7% isopropyl alcohol and scented with 10% Simple Green. Animals were transported to the context in squads of four using a square black tub divided into four compartments with a plastic insert and filled with bedding and covered with a wooden lid.

### Context B

The context B environment consisted of aluminum (side walls) and clear Plexiglas (front and top) chambers (30 cm × 25 cm × 25 cm, Med-Associates, Inc. St. Albans, VT, USA). The rear wall was white opaque plastic and the distinct grid flooring pattern consisted of two planes of up/down “staggered” stainless steel rods (4.8 mm thick) spaced 1.6 cm apart (center to center; Med-Associates, Inc. St. Albans, VT, USA). The background fan was turned off. The context B chambers were cleaned with 10% ethanol and scented with 10% Windex. Animals were transported to the context in squads of four in their individual home cages, which were slid onto hanging racks mounted to a portable cart and covered with a white cloth sheet.

### Cues, Training and Testing

For auditory cue fear conditioning, rats received delay conditioning using four tone—shock pairing [Baseline (BL) = 2 min, CS = 2800 Hz; Pure tone; 77 dB; 30 s each, US = 0.8 mA; 2 s each; co-terminating with the tone CS, Inter trial *interval*_(ITI)_ = 2 min, end period = 2 min]. Freezing was scored during the CS presentations on the fear conditioning day. Based on the fear level to the last CS of the fear conditioning day, rats were then rank ordered and assigned to experimental groups in a randomized block order to match the groups for average freezing. Seventy two hours after fear conditioning the retrieval and/or extinction training procedure was conducted. Seventy two hours interval was used because the original study on memory deconsolidation (Myers et al., 2006) found that extinction trained at an interval of 72 h following fear acquisition (long-interval extinction) was sensitive to disruption through reinstatement, renewal, and spontaneous recovery when compared to that of 24 h.

Rats were divided into five groups: (1) Retrieval before extinction (Ret-Ext)- 3 CS-alone massed tones (5 s ITI) were presented for fear retrieval in context B, after which the rats were taken back to the home cage for 10 min and then extinction training session consisting of 50 CS-alone massed presentations (5-s ITI), was performed in the same context. Our procedure was similar to Monfils et al. (2009) procedures except that we used 3 CSs, rather than 1, for retrieval; (2) Extinction before retrieval (Ext-Ret)–Behavioral procedures and retrieval sessions were same as Ret-Ext except that retrieval was given 10 min after the extinction session; (3) Normal Extinction (Normal Ext)–No retrieval was given. The extinction training session consisted of 53 massed CS-alone presentations (5-s ITI) in Context B. Note that the total number of CSs presented was equal to the number in the retrieval groups; (4) Immediate extinction (Immediate Ext) rats were trained in Context A and underwent extinction in Context B 10 min after training using the same parameters of the Normal-Ext group. Our procedure was similar to Maren and Chang (2006) and Myers et al. (2006) in which immediate extinction has been shown to elicit memory attenuation effects under some conditions; and (5) No extinction (No Ext) – Fear conditioned rats were exposed to the B context but no retrieval or extinction session was given. However, during the testing stage, they received tone test like all other groups. This group served as a fear memory retention control. When animals received the retrieval or extinction, the BL period was always 2 min (i.e., 2 min after placing the animals in context B, they received tones).

We used a 10 min interval between retrieval/reminder and extinction sessions based on previous studies (Myers et al., 2006; Monfils et al., 2009). We used three CSs to reactivate the memory instead of one. This is because the first CS typically elicited only about 40–50% freezing behavior in our rats during a typical extinction session. However, subsequent 2nd and 3rd retrieval CSs gave rise to higher freezing behavior (~70–90%). Based on this observation and in order to fully activate all aspects of a fear memory, we decided to use a total of 3 CSs instead of 1 CS for our reconsolidation or retrieval experiments. In the first experiment, we tested the extinction memory 24 h after the extinction training procedure in Context B. Other than using a different context and omitting the US, all the test sessions were conducted the same as fear conditioning. A second test was given 21 days after the extinction session to measure the spontaneous recovery of fear in context B.

In the second experiment, we used the same training parameters as described for experiment 1, however, for half of the rats, testing was done 3 h and 15–19 min after the various extinction procedures or about 4 h after retrieval, so that they were all tested within the typical 6 h consolidation/reconsolidation window (Nader et al., 2000). For the other half of the rats, testing was done 24 h after the various extinction procedures.

### Dependent Measure

For all experiments, freezing was the index of fear memory. We used a commercially available near-infra data acquisition system and software (Med Associates Video Freeze) that had been calibrated to very experienced human observers. Freezing is defined as the absence of all visible movement except that required for respiration.

### Statistical Analyses

Data were statistically analyzed using between-subjects analysis of variance (ANOVAs) and repeated measures (RM) ANOVAs where appropriate. Fear acquisition and extinction data were analyzed using RM (trial, bin) ANOVAs. BL freezing and average freezing during the tone test were analyzed separately by one-way ANOVAs. *Post hoc* comparisons were performed following significant findings using a Tukey’s multiple comparisons for two-way RM ANOVA and for one-way ANOVA. The level of significance used for all analyses was *P* < 0.05.

## Results

### Long-Term Extinction Memory After Ret-Ext, Ext-Ret and Immediate Ext Procedures

**Figure 1:**
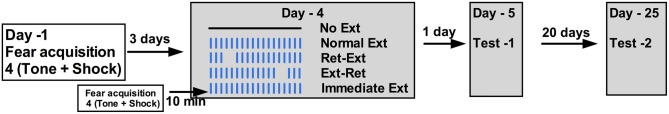
**Schema of Experiment 1.** Tone; 77dB; 30 s each, Shock = 0.8 mA; 2 s each, Inter trial interval (ITI) = 2 min during fear conditioning in context A. Fear conditioning was done on Day-1 and extinction, test 1 and test 2 were done on Day-4, 5 and 25 respectively. On extinction day in context B, Ret-Ext and Ext-Ret groups received a retrieval session consisting of three massed tones, 5 s ITI and extinction session consisted of 50 massed tones, 5 s ITI. Retrieval and extinction sessions were 10 min apart.

In experiment 1, fear conditioned rats received various extinction procedures and were tested at 1 and 21 days after for spontaneous recovery of fear (schema of experiment in Figure 1).

**Figure 2:**
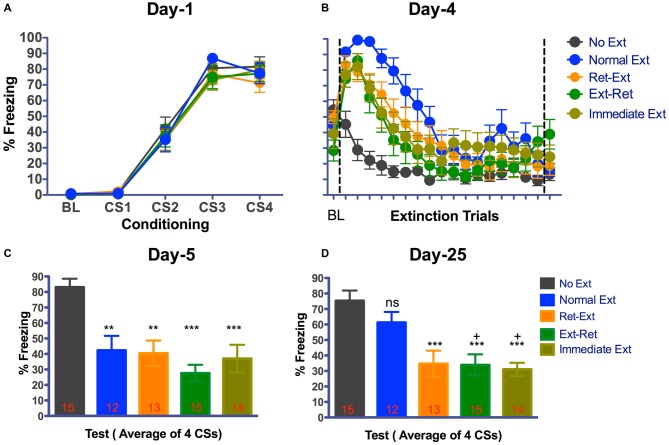
**Attenuation of fear memory after various extinction procedures.** Graphs show fear and extinction learning curves **(A,B)** and extinction memory during a memory test carried out 1 and 21 day(s) after the end of each extinction procedure in context B **(C,D)**. Mean ± SEM freezing during Baseline (BL) and tone for all rats were measured. **(A)** Fear acquisition. Rats were fear conditioned with 4 tone-shock pairings on Day-1 in context A. All animals acquired cue fear. **(B)** Fear retrieval and extinction. Rats received retrieval and extinction procedures in context B on Day-4. Immediate extinction group was fear conditioned in Context A with four tone-shock pairings and 10 min later they received normal fear extinction session in context B. Each data point for extinction trials represent the average of 3 CSs except the 17th data point that represents average of 2 CSs totaling 53 CSs. Data point after the line break represent the average of 3 retrieval CSs for Ret-Ext and Ext-Ret groups. All rats showed significant within session extinction learning on Day-4. **(C)** Fear extinction memory test. As a measure of extinction memory, rats received 4 tone presentations in context B on Day-5. **(D)** Spontaneous recovery test. Rats received four tone presentations in context B on Day-26. Normal Ext group showed freezing similar to No Ext group suggesting significant spontaneous recovery of fear. Ret-Ext, Ext-Ret and Immediate Ext groups showed no spontaneous recovery of fear. One-Way ANOVA with Tukey’s multiple comparison test was used for Days-5 and 26. (ns, not significant; **P < 0.01; ***P < 0.001 vs. No Extinction **(C,D)**. ^+^*P* < 0.05 vs. Normal Extinction **(D)**. Groups: No Ext, no extinction; Normal Ext, normal extinction; Ret Ext, retrieval first + extinction; Ext—Ret, extinction + retrieval later; Immediate Ext, Fear conditioning first + immediate extinction. Rats were tested repeatedly on Day-5 and Day-26.

Figure 2A shows fear acquisition data from different groups of rats. All rats developed significant tone fear during acquisition (*F*_(3,192)_ = 267.79, *P* < 0.0001 *n* = 69) in context A. The main effect of group (*F*_(4 64)_ = 0.39, *P* = 0.8115) or interaction (*F*_(12,192)_ = 0.44, *P* = 0.9451) was not significant. Animals were then divided into four groups: (Ret-Ext (*n* = 13); Ext-Ret (*n* = 15); Normal Ext (*n* = 12); and No Ext (*n* = 15)) on the basis of their freezing levels to last CS during fear conditioning to ensure that groups were balanced (see the “Materials and Methods” Section for more details). Since rats in Immediate Ext (*n* = 14) group underwent fear extinction 10 min after fear conditioning, we were unable to balance in advance. However, Immediate Ext group acquired fear that was similar to all other groups (Figure 2A). Freezing during retrieval (3 CSs) and extinction sessions (50 or 53 CSs) are shown in Figure 2B as one graph but the sessions were conducted 10 min apart for the groups Ret-Ext and Ext-Ret. Extinction and subsequent tests were done in context B. Each data point for extinction trials in Figure 2B represent average of 3 CSs except the 17th data point that represents average of 2 CSs totaling 53 CSs. BL fear did not differ among the groups (one way-ANOVA *F*_(4,64)_ = 2.212, *P* = 0.0775). All rats acquired significant fear extinction reduction across extinction/retrieval sessions (*F*_(17,1088)_ = 64.61, *P* < 0.0001). The main effect of group (*F*_(4,64)_ = 5.07, *P* = 0.0013) and interaction (*F*_(68,1088)_ = 3.84, *P* < 0.0001) were significant. Note that rats belonging to No Ext group were simply exposed to context B but were not presented with any tone (Figure 2A) on the extinction day. Initial freezing in No Ext group was fear that generalized from the conditioning context and extinguished over time in context B. All extinction groups showed significant fear during initial stages of extinction session when compared to No Ext group. There were no significant differences between extinction groups and No Ext group during final stages of extinction session (for details, see Table 1).

**Table 1:** **Fear extinction data analysis for experiment 1**.

	CS1		CS9		CS18	
Comparison	*F*_(1,1152)_	*P*	*F*_(1,1152)_	*P*	*F*_(1,1152)_	*P*
No Ext vs. Ret-Ext	**5.106**	**0.0029**	2.256	0.5008	0.7349	0.9854
No Ext vs. Immediate Ext	**4.34**	**0.0187**	0.9451	0.9631	1.54	0.8124
No Ext vs. Ext-Ret	**4.069**	**0.0333**	0.08042	>0.9999	3.575	0.0853
No Ext vs. Normal Ext	**6.086**	**0.0002**	1.843	0.6894	0.3862	0.9988
Ret-Ext vs. Immediate Ext	0.8362	0.9764	1.307	0.8875	0.7626	0.9832
Ret-Ext vs. Ext-Ret	1.186	0.9186	2.178	0.5364	2.71	0.3093
Ret-Ext vs. Normal Ext	1.054	0.9457	0.3524	0.9991	0.322	0.9994
Immediate Ext vs. Ext-Ret	0.3423	0.9992	0.8661	0.9731	1.973	0.6311
Immediate Ext vs. Normal Ext	1.892	0.6678	0.9214	0.9664	1.074	0.942
Ext-Ret vs. Normal Ext	2.25	0.5036	1.767	0.7222	2.984	0.2164

*Extinction data for experiment 1 were analyzed using RM (trial, bin) ANOVAs (Figure 2B). All rats showed significant within session extinction learning (Day-4). Further analysis of fear extinction data was done using corrected Tukey’s multiple comparison test. Percentage freezing for CS1, CS9 and CS18 were presented in this table instead of presenting all the CSs. Each data point for extinction trials represent average of 3 CSs except the 17th data point that represents average of 2 CSs totaling 53 CSs*.

On days-5 and 25 (1 and 21 day(s) after the various extinction procedures), extinction memory tests were done in context B using the same protocol used for fear conditioning (minus the shocks; Figures 2C,D). Freezing for 4 CSs was averaged. On Day 5 test, BL fear did not differ (one way-ANOVA *F*_(4,64)_ = 1.296, *P* = 0.2813) among the groups (average percent Freezing—Ret-Ext = 15.51 ± 6.246, Ext-Ret = 2.740 ± 1.308, Normal Ext = 12.42 ± 5.1, Immediate Ext = 8.778 ± 4.0 and No Ext = −10.21 ± 3.639). On day 5 test, average percent freezing of No Ext group was larger than 80, which was similar to initial freezing level of all other groups during the extinction session on day 4 (Figure 2C). These data indicate that there is no fear expression impairments on Day-5. Day-5 test revealed a significant extinction memory (one way-ANOVA *F*_(4,64)_ = 9.241, *P* < 0.0001) in Ret-Ext (*P* < 0.01), Ext-Ret (*P* < 0.001), Normal Ext (*P* < 0.01) and Immediate Ext groups (*P* < 0.001) when compared to No Ext group (Figure 2C).

On Day 25 test, BL fear did not differ (one way-ANOVA *F*_(4,64)_ = 2.323, *P* = 0.0663) among the groups (Ret-Ext = 25.11 ± 3.681, Ext-Ret = 11.71 ± 2.182, Normal Ext = 15.60 ± 5.304, Immediate Ext = 15.47 ± 4.093 and No Ext = −23.50 ± 3.968). On day-25 test, average percent freezing of No Ext group was similar to freezing on day-4 suggesting no fear memory retrieval/expression issues on Day-25 (Figure 2D) from our No Ext group. As expected, the Day-25 test revealed a significant spontaneous recovery of fear (one way-ANOVA *F*_(4,64)_ = 9.099, *P* < 0.0001), in the Normal Ext group and percent freezing was not different from the No Ext group (*P* > 0.05, Figure 2D). However, Ret-Ext, Ext-Ret and Immediate Ext groups showed very little freezing (all three groups *P* < 0.001) when compared to No Ext group (Figure 2D) Interestingly, Ext-Ret (*P* < 0.05) and Immediate Ext (*P* < 0.05) groups showed lower fear than the Normal Ext group on Day-25 test. The Ret-Ext group also showed low fear compared to the Normal Ext group, however the difference fell short of statistical reliability (*P* = 0.0733). In general, these results are consistent with a retrieval model, in that extinction retention was facilitated by each of the procedures that differed from the standard extinction method.

### Short-Term Fear Memory After Successful Within Session Extinction in Ret-Ext Group

**Figure 3:**
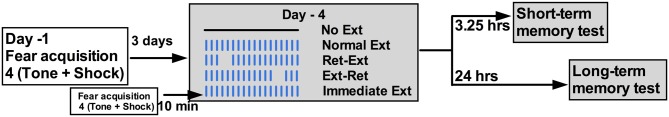
**Schema of Experiment 2.** Tone; 77dB; 30 s each, Shock = 0.8 mA; 2 s each, Inter trial interval (ITI) = 2 min during fear conditioning in context A. Fear conditioning was done one Day-1 and extinction was done on Day-4. Short-term test was done 3.25 h after extinction procedures on Day-4. Long-term test was done 24 h after extinction procedures on Day-5. On extinction day in context B, Ret—Ext and Ext—Ret groups received a retrieval session consisted of 3 massed tones, 5 s ITI and extinction session consisted of 50 massed tones, 5 s ITI. Retrieval and extinction sessions were 10 min apart.

Both Myers et al. (2006) and Monfils et al. (2009) timed their extinction sessions to coincide with a period where cellular memory consolidation processes are assumed to be ongoing. This is based on classic consolidation studies suggesting that there is a period that starts after encoding and persisting for up to about 6 h during which memory is vulnerable to manipulations such as electroconvulsive shock or protein synthesis inhibitors (e.g., Agranoff et al., 1965; McGaugh, 1966). One characteristic of studies that disrupt consolidation and reconsolidation is that the loss of memory does not happen immediately but rather appears during a long-term memory test 24 h or more hours later (Nader et al., 2000; Schafe and LeDoux, 2000). When memory was tested shortly after anisomycin delivery, auditory fear conditioning was intact (Nader et al., 2000; Schafe and LeDoux, 2000). Therefore, if immediate extinction and reminder-extinction treatments affect consolidation they too should leave short-term fear performance intact and deficits should emerge only at long-term test points. Therefore to diagnose this pattern we conducted a short-term test of extinction memory 3 h and 25 min after extinction (Figure 3). This also ensured that the interval between the retrieval treatment in the Ret-Ext group also fell within the 4 h window used by Schafe and LeDoux (2000) and Nader et al. (2000).

**Figure 4:**
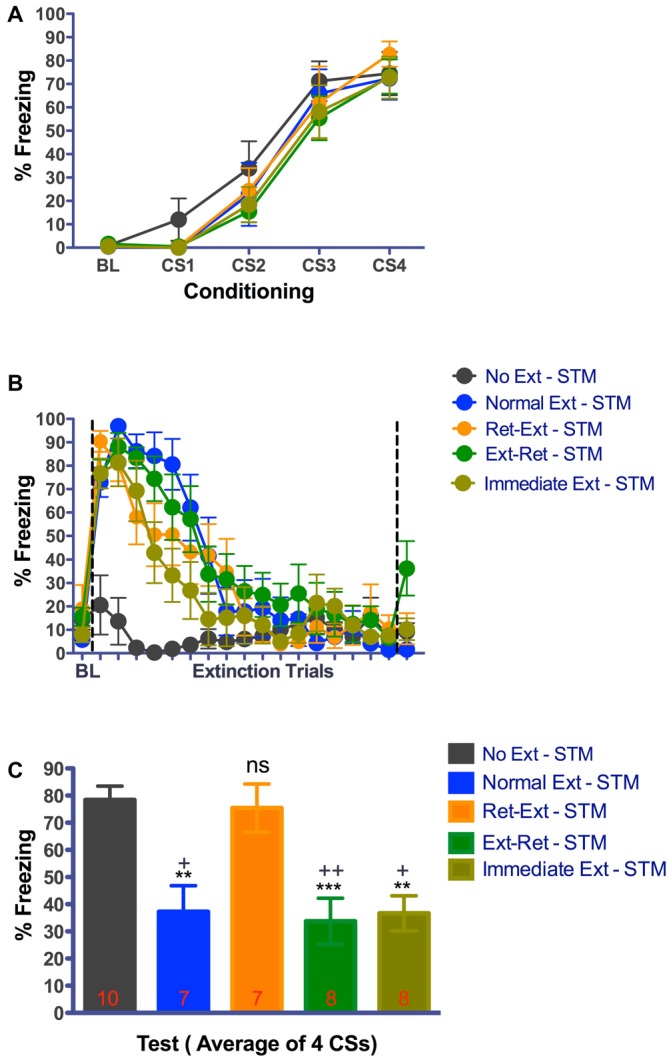
**Rats failed to show extinction memory 3.25 h after successful retrieval—extinction session. (A)** Fear acquisition. Rats were fear conditioned with four tone-shock pairings on Day-1 in context A. All animals acquired cue fear. **(B)** Fear retrieval and extinction. Rats received retrieval and extinction procedures in context B on Day-4. Immediate extinction group was fear conditioned in Context A with four tone-shock pairing and 10 min later they received normal fear extinction session in context B. All groups showed significant within session extinction learning on Day-4. Each data point for extinction trials represent average of 3 CSs except the 17th data point that represents average of 2 CSs totaling 53 CSs. Data point after the line break represent the average of 3 retrieval CSs for Ret-Ext and Ext-Ret groups. **(C)** Short-term fear extinction memory test. Rats received four tone only presentations in context B 3.25 h after the extinction procedures. Normal Ext, Ext-Ret, Immediate Ext groups showed significant low fear memory where as Ret-Ext group showed no traces of extinction memory in this test. Extinction during reconsolidation did not cause immediate memory erasure. One-Way ANOVA with Tukey’s multiple comparison test was used for Day-4. (ns, not significant; **P < 0.01; ***P < 0.001 vs. No Extinction **(C)**. ^+^*P* < 0.05; ^++^*P* < 0.01 vs. Ret-Ext. Groups: No Ext, no extinction; Normal Ext, normal extinction; Ret-Ext, retrieval first + extinction; Ext-Ret, extinction + retrieval later; Immediate Ext, Fear conditioning first + immediate extinction. To avoid any potential confounding effect of the test by itself, rats tested in short-term memory after extinction were not used on 24 h memory test.

**Figure 5:**
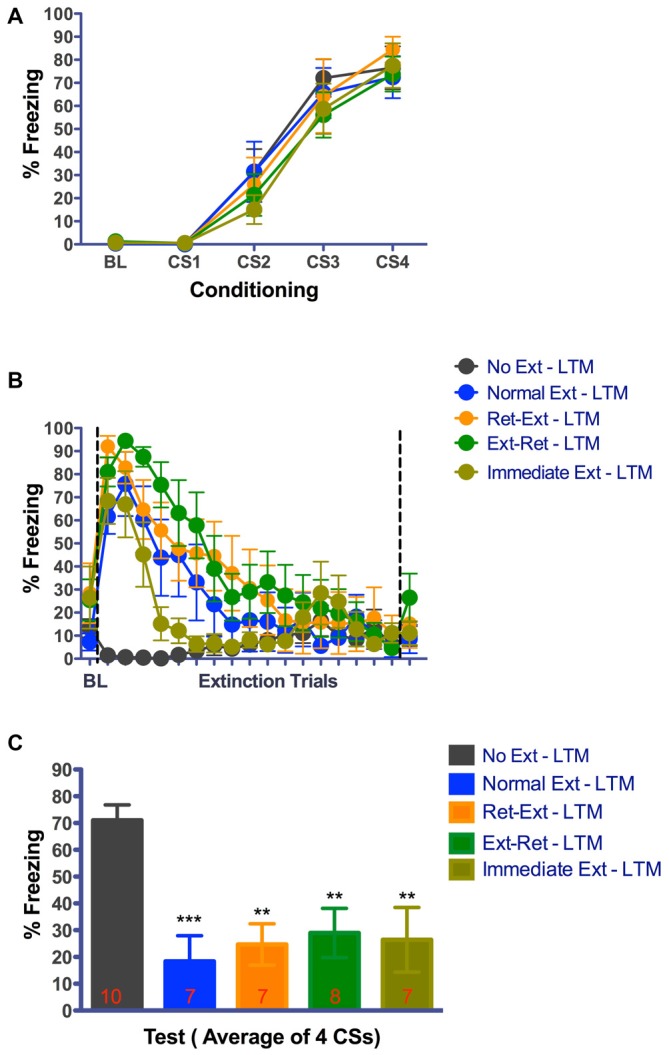
**Good extinction memory 24 h after successful retrieval—extinction session. (A)** Fear acquisition. Rats were fear conditioned with four tone-shock pairings on Day-1 in context A. All animals acquired cue fear. **(B)** Fear retrieval and extinction. Rats received retrieval and extinction procedures in context B on Day-4. Immediate extinction group was fear conditioned in Context A with four tone-shock pairings and 10 min later they received normal fear extinction session in context B. All groups showed significant within session extinction learning on Day-4. Each data point for extinction trials represent average of 3 CSs except the 17th data point that represents average of 2 CSs totaling 53 CSs. Data point after the line break represent the average of 3 retrieval CSs for Ret-Ext and Ext-Ret groups. **(C)** Long-term fear extinction memory test. Rats received four tone only presentations in context B 24 h after the extinction procedures. Normal Ext, Ret-Ext, Ext-Ret, Immediate Ext groups showed significant low fear memory. One-Way ANOVA with Tukey’s multiple comparison test was used for Day-4.) (**P < 0.01, ***P < 0.001 vs. No Ext). Groups: No Ext, no extinction; Normal Ext, normal extinction; Ret-Ext, retrieval first + extinction; Ext-Ret, extinction + retrieval later; Immediate Ext, Fear conditioning first + immediate extinction. To avoid any potential confounding effect of the test by itself, different groups of rats were tested in the short-term memory and 24 h memory tests.

As shown in Figures 4A, 5A, all rats acquired significant tone fear across acquisition trials (Figure 4A, *F*_(3,105)_ = 106.96, *P* < 0.0001, *n* = 40; Figure 5A, *F*_(3,102)_ = 110.67, *P* < 0.0001, *n* = 39) in context A. In Figure 4A, the main effect of group (*F*_(4,35)_ = 0.62, *P* = 0.6501) or interaction (*F*_(12,105)_ = 0.32, *P* = 0.9839) was not significant. In Figure 5A, the main effect of group (*F*_(4,34)_ = 0.34, *P* = 0.8496) or interaction (*F*_(12,102)_ = 0.40, *P* = 0.9608) was also not significant. Animals were equally split into two sets of groups (Ret-Ext, Ext-Ret, Normal Ext and No Ext) on the basis of their final levels of fear, ensuring that groups were balanced before extinction. Since, rats in Immediate Ext group underwent fear extinction 10 min after fear conditioning, we were unable to balance fear levels in advance. However, as shown in Figures 4A, 5A, Immediate Ext group acquired fear that was similar to all other groups. Freezing during retrieval and extinction sessions is shown in Figures 4B, 5B. BL fear did not differ among the groups (Figure 4B one way-ANOVA *F*_(4,36)_ = 1.142, *P* = 0.3523; Figure 5B one way-ANOVA *F*_(4,35)_ = 1.470, *P* = 0.2323). All rats acquired significant fear extinction across extinction trials as shown in Figure 4B (*F*_(17,595)_ = 50.01, *P* < 0.0001) and Figure 5B (*F*_(17,578)_ = 29.50, *P* < 0.0001) in context B. In Figure 4B, the main effect of group (*F*_(4,35)_ = 6.13, *P* = 0.0008) and interaction (*F*_(68,595)_ = 5.38, *P* < 0.0001) were significant. In Figure 5B, the main effect of group (*F*_(4,34)_ = 5.47, *P* = 0.0016) and interaction (*F*_(68,578)_ = 4.80, *P* < 0.0001) were significant. All extinction groups showed significant fear during initial stages of extinction session when compared to No Ext group. There were no differences between extinction groups and No Ext group during final stages of extinction session (for details, see Tables 2, 3). Note that rats in the No Ext group were simply exposed to context B but were not presented with any tone while, the other groups received repeated tone presentations. Initial freezing in the No Ext group was fear that generalized from the conditioning context and extinguished over time in context B.

**Table 2:** **Fear extinction data analysis of short-term memory (STM) groups for experiment 2**.

	CS1		CS9		CS18	
Comparison	*F*_(1,630)_	*P*	*F*_(1,630)_	*P*	*F*_(1,630)_	*P*
No Ext-STM vs. Normal Ext-STM	**6.332**	**<0.0001**	1.43	0.8503	0.9208	0.9664
No Ext-STM vs. Ret-Ext-STM	**8.45**	**<0.0001**	1.146	0.9275	0.1553	>0.9999
No Ext-STM vs. Ext-Ret-STM	**7.023**	**<0.0001**	2.574	0.3629	3.394	0.1167
No Ext-STM vs. Immediate Ext-STM	**7.051**	**<0.0001**	1.275	0.8962	0.1304	>0.9999
Normal Ext-STM vs. Ret-Ext-STM	1.952	0.6406	0.2617	0.9997	0.9921	0.9561
Normal Ext-STM vs. Ext-Ret-STM	0.4074	0.9985	0.9975	0.9553	3.987	0.0397
Normal Ext-STM vs. Immediate Ext-STM	0.4326	0.9981	0.1925	>0.9999	0.9963	0.9555
Ret-Ext-STM vs. Ext-Ret-STM	1.609	0.7866	1.268	0.8982	2.962	0.2236
Ret-Ext-STM vs. Immediate Ext-STM	1.583	0.7962	0.0778	>0.9999	0.02834	>0.9999
Ext-Ret-STM vs. Immediate Ext-STM	0.026	>0.9999	1.232	0.9075	3.096	0.1852

*Extinction data for experiment 2 were analyzed using RM (trial, bin) ANOVAs (Figure 4B). All rats showed significant within session extinction learning (Day-4). Further analysis of fear extinction data was done using corrected Tukey’s multiple comparison test. Percentage freezing for CS1, CS9 and CS18 were presented in this table instead of presenting all the CSs. Each data point for extinction trials represent average of 3 CSs except the 17th data point that represents average of 2 CSs totaling 53 CSs*.

**Table 3:** **Fear extinction data analysis of long-term memory (LTM) groups for experiment 2**.

	CS1		CS9		CS18	
Comparison	*F*_(1,612)_	*P*	*F*_(1,612)_	*P*	*F*_(1,612)_	*P*
No Ext-LTM vs. Normal Ext-LTM	**6.755**	**<0.0001**	1.182	0.9195	0.1464	>0.9999
No Ext-LTM vs. Ret-Ext-LTM	**10.16**	**<0.0001**	2.744	0.2971	0.6031	0.9931
No Ext-LTM vs. Ext-Ret-LTM	**9.273**	**<0.0001**	2.674	0.3235	1.941	0.6455
No Ext-LTM vs. Immediate Ext-LTM	**7.515**	**<0.0001**	0.2277	0.9998	0.1434	>0.9999
Normal Ext-LTM vs. Ret-Ext-LTM	3.143	0.1727	1.44	0.8468	0.691	0.9884
Normal Ext-LTM vs. Ext-Ret-LTM	2.067	0.5879	1.325	0.8824	1.919	0.6557
Normal Ext-LTM vs. Immediate Ext-LTM	0.7004	0.9878	0.8795	0.9716	0.2673	0.9997
Ret-Ext-LTM vs. Ext-Ret-LTM	1.179	0.92	0.1625	>0.9999	1.205	0.914
Ret-Ext-LTM vs. Immediate Ext-LTM	2.443	0.4178	2.32	0.4721	0.4238	0.9982
Ext-Ret-LTM vs. Immediate Ext-LTM	1.344	0.877	2.234	0.5113	1.643	0.7733

*Extinction data for experiment 2 were analyzed using RM (trial, bin) ANOVAs (Figure 5B). All rats showed significant within session extinction learning (Day-4). Further analysis of fear extinction data was done using corrected Tukey’s multiple comparison test. Percentage freezing for CS1, CS9 and CS18 were presented in this table instead of presenting all the CSs. Each data point for extinction trials represent average of 3 CSs except the 17th data point that represents average of 2 CSs totaling 53 CSs*.

At 3.25 h test, freezing was significantly different between the groups (one way-ANOVA *F*_(4,39)_ = 9.270, *P* < 0.0001). Despite successful within session extinction the Ret-Ext group showed a robust short-term fear memory (3.25 h) in the extinction context that was not statistically different from No Ext group (*P* > 0.05, Figure 4C). Note that freezing was almost identical in No Ext (~78%) and Ret-Ext (~75%) groups. At 3.25 h test, this high fear in Ret-Ext was significantly different from Ext-Ret (*P* < 0.01), Normal Ext (*P* < 0.05) and Immediate Ext (*P* < 0.05). However, the Normal Ext (*P* < 0.01), Ext-Ret (*P* < 0.001) and Immediate Ext (*P* < 0.01) groups showed a significant short-term extinction memory at 3.25 h when compared to No Ext group. Replicating Experiment 1, in Experiment 2 at 24 h test, Ret-Ext, Ext-Ret, Immediate Ext and Normal Ext groups showed significant extinction memory (one way-ANOVA *F*_(4,39)_ = 6.910, *P* < 0.0003) when compared to No Ext group (Figure 5C–Normal Ext *P* < 0.001 and rest of the groups *P* < 0.01 when compared to No Ext group).

## Discussion

Following auditory fear conditioning we evaluated the efficacy of several extinction protocols relative to a standard massed training extinction protocol consisting of 53 presentations of the CS spaced 5 s apart. The standard extinction protocol caused significant loss of fear when the rats were tested 1 day after extinction, confirming earlier studies using similar protocols (Cain et al., 2003). As expected there was a significant return of fear when the test occurred 21 days after extinction. However, this spontaneous recovery was not observed in our three modified extinction protocols. Similar to Myers et al. (2006), we found that extinguishing fear shortly after fear conditioning defeated spontaneous recovery (for opposite results in both rodents and humans, see Chan et al., 2010; Costanzi et al., 2011; Kindt and Soeter, 2013; Stafford et al., 2013). Similar to Monfils et al. (2009) presenting a few CSs prior to the start of the regular extinction session also prevented spontaneous recovery (for similar results in both rodents and humans, see Schiller et al., 2010; Clem and Huganir, 2010; Rao-Ruiz et al., 2011; Agren et al., 2012). According to Monfils et al.’s (2009), reconsolidation update hypothesis which was confirmed at the molecular to systems level, placement of brief CS presentations prior to extinction is critical in that they are hypothesized to open a window of memory vulnerability that allows the subsequent extinction session to erase the original fear memory. However, we found that the order of a retrieval session and an extinction session made little difference as spontaneous recovery was also reduced when the ordering of the short and longer sessions was reversed. Our results are consistent with findings of previous articles that used similar Ext- Ret approach in adolescent rats (Baker et al., 2013) and alcoholic beer memory retrieval in adult rats (Millan et al., 2013). The reconsolidation update hypothesis does not anticipate such a result. As a potential alternative that is consistent with retrieval views of memory (Bouton, 1993; Bjork, 1994), we suggest that having two different types of extinction sessions close in time makes the extinction memory more retrievable and thereby reduces spontaneous recovery of fear by making the extinction better able to interfere with retrieval of the original fear memory. However, it is possible that the Retrieval-Extinction and Extinction-retrieval procedures produce their effects via different mechanisms. The Extinction-Retrieval effect could be caused because two different extinction sessions lead to better retrieval of the extinction memory. The Retrieval-Extinction effect may be caused by a true deconsolidation of the original fear memory. This is supported by the finding that the fear memory was intact at the short-term test for the Retrieval-Extinction procedure but not the Extinction-Retrieval procedure.

### Deconsolidation

Both the Myers et al. (2006) immediate extinction and the Monfils et al. (2009) reminder-extinction accounts suggest that the original fear memory is erased when extinction occurs during a period when memory has been destabilized. For immediate extinction this vulnerability is because the fear memory has not yet consolidated. For retrieval-extinction, reminding the animal of the CS opens a period of vulnerability during which a memory must be reconsolidated where the original fear memory is replaced by a new extinction memory and thus the original memory no longer exists. These ideas are based on the finding that memory is lost when a protein synthesis inhibitor is administered during these windows. Interestingly, with the protein synthesis inhibitor fear memory is intact when the CS is tested shortly (e.g., 6 h or less) after the amnestic manipulation but memory degrades after that with amnesia being observed 24 h later (Nader et al., 2000; Schafe and LeDoux, 2000). Such a finding is a fundamental aspect of consolidation and reconsolidation theory as it provides evidence that memory stabilization rather than memory expression is affected by the amnestic treatment. Therefore, we also tested memory retrieval shortly after our extinction manipulations. If the manipulation affected memory stabilization then extinction memory should be intact and fear levels high during this test. The only procedure that showed this pattern was the retrieval-extinction protocol as short-term fear memory was abated with the three other procedures. The data suggest that the retrieval-extinction order works via a mechanism that is distinct from the other procedures and the pattern observed is quite consistent with reconsolidation theory. Based on our short-term memory (STM) results, we conclude that Ret-Ext group might update extinction memory with safety information and this mechanism could explain our results. Interestingly using contextual fear conditioning procedure, Rao-Ruiz et al. (2011) reported that brief un-reinforced recall of contextual fear memory lead to initial synaptic depression and endocytosis of GluA1, A2 and A3 containing AMPAR expression within 1–4 h. In the same experiment, they found a subsequent increase in synaptic strength and increase in GluA2 containing AMPARs in the synapse at 7 h. However, high fear memory in our STM test 3.15 h after retrieval-extinction procedure, was not in parallel to the biochemical findings of Rao-Ruiz et al. (2011) that showed the hippocampal synaptic changes immediately (1–4 h) after retrieval. Using normal extinction, extinction- retrieval and immediate extinction procedures, we found results similar to the studies using normal extinction (e.g., studies that tested the memory at short-term interval <6 h; Quirk, 2002; Berman et al., 2003; but see Archbold et al., 2013 for opposite results) suggesting normal extinction learning dependent inhibition results in expression of extinction memory.

### Immediate Extinction

Like Myers et al. (2006), we found that starting extinction shortly after training produced an effective loss of fear in that there was little spontaneous recovery of fear 3 weeks after extinction. While Myers et al. (2006) suggested this was caused by a disruption of memory consolidation the fact that fear was absent at the short-term test raises the possibility that fear expression rather than consolidation was affected. Such a pattern is readily explained by the ambiguity theory of Bouton (1993). Bouton (1993) suggests that a memory for both an acquisition episode and an extinction episode are formed and fear expression is determined by which episode is recalled at the time of test. When acquisition and extinction occur at the same time the subject may concatenate the two treatments into a single episode that is dominated by the extinction memory. If this happens extinction recall should be robust regardless of when memory is tested.

In contrast to Myers et al. (2006) and the results reported here, Maren and Chang (2006) found that giving extinction immediately after fear conditioning results in very poor loss of fear (see also studies in rats (Morris et al., 2005; Archbold et al., 2010), mice (Stafford et al., 2013) and human (Alvarez et al., 2007; Norrholm et al., 2008; Schiller et al., 2008; Huff et al., 2009) for failure of immediate extinction on fear memory). This failure of an immediate extinction procedure appears to be caused by strong and persistent stress or fear that continues after the fear acquisition session (Maren, 2014). The levels of BL fear prior to immediate extinction appear to be considerably lower in our study than in Maren and Chang (2006) and Chang and Maren (2009). In those studies the rats froze about 80% prior to CS presentation, while BL freezing in ours was 40% or less (Figures 2B, 4B, 5B). This BL difference occurred despite similar levels of CS elicited freezing in both labs. We used very different contexts and having distinct acquisition and extinction contexts likely caused an overall reduction in fear and anxiety during extinction with our procedures.

The diversity of findings found with parametric manipulations of extinction such as those reported here open up more questions on long and short-term dynamic aspect of fear memory reconsolidation and retrieval. Based on our results we conclude that both retrieval and reconsolidation processes contribute to long-term extinction memories. The degree to which they contribute depends on experimental procedures. All designs except the Ret-Ext appear to primarily reflect retrieval processes. Ret-Ext seems unique in that the short-term fear memory remains intact after extinction; much as it does in classic consolidation and reconsolidation studies using amnestic agents. Since there is need to develop more effective interventions, studies exploring both short-term and long-term performance may benefit translation of pre-clinical results to clinical settings.

## Author Contributions

AMP and JS designed the research, analyzed the data. MM, JH, P-KO’N and DW performed the research. RP designed the research, performed the research, analyzed the data and wrote the article. MSF designed the research, analyzed the data and wrote the article. IZ Performed the research and analyzed the data.

## Conflict of Interest Statement

The authors declare that the research was conducted in the absence of any commercial or financial relationships that could be construed as a potential conflict of interest.
